# Altered retinal vasculature in childhood cancer survivors: Data from the German CVSS‐study

**DOI:** 10.1111/aos.17438

**Published:** 2025-01-23

**Authors:** Alexander K. Schuster, Anna Maria Voigt, Tamara Jäger, Stefan Nickels, Andreas Schulz, Jörg Faber, Arthur Wingerter, Hiltrud Merzenich, Irene Schmidtmann, Manfred E. Beutel, Thomas Münzel, Karl J. Lackner, Norbert Pfeiffer, Philipp S. Wild

**Affiliations:** ^1^ Department of Ophthalmology University Medical Center Mainz Mainz Germany; ^2^ Preventive Cardiology and Preventive Medicine, Center for Cardiology University Medical Center of the Johannes Gutenberg‐University Mainz Mainz Germany; ^3^ Department of Pediatric Hematology/Oncology/Hemostaseology University Medical Center of the Johannes Gutenberg University Mainz Mainz Germany; ^4^ Department of Biomedical Statistics University Medical Center of the Johannes Gutenberg‐University Mainz Mainz Germany; ^5^ Department of Psychosomatic Medicine and Psychotherapy University Medical Center Mainz Mainz Germany; ^6^ Center for Cardiology University Medical Center of the Johannes Gutenberg‐University Mainz Mainz Germany; ^7^ Center for Thrombosis and Hemostasis University Medical Center of the Johannes Gutenberg‐University Mainz Mainz Germany; ^8^ Institute for Clinical Chemistry and Laboratory Medicine University Medical Center of the Johannes Gutenberg‐University Mainz Mainz Germany; ^9^ DZHK (German Center for Cardiovascular Research) Partner Site Rhine‐Main Mainz Germany; ^10^ Systems Medicine Institute of Molecular Biology (IMB) Mainz Germany

**Keywords:** arteriovenous ratio, central retinal arteriolar equivalent, central retinal venular equivalent, chemotherapy, childhood cancer survivors, radiotherapy

## Abstract

**Aims:**

Childhood cancer is a risk factor for cardiovascular diseases in later life. Retinal examination allows to non‐invasively observe the vasculature of an end‐organ. We observe alterations in long‐term childhood cancer survivors (CCS).

**Methods:**

In the Cardiac and Vascular Late Sequelae in Long‐Term Survivors of Childhood Cancer‐Study, 1002 CCS (23–48 years) having neoplasia prior to 15 years of age were prospectively enrolled in a general and ophthalmologic examination including fundus photography. Central retinal vessel equivalents (arterial: CRAE; venous CRVE) were measured and linear regression analysis was computed to compare CCS to controls from the population‐based Gutenberg Health Study (GHS) with adjustment for potential cardiovascular and ophthalmological confounders. Differences in cancer types and treatments were explored.

**Results:**

For 837 CCS (45.3% female), CRAE and CRVE were conducted. Both were smaller in CCS previously having leukaemia, central nervous system tumour, neuroblastoma, renal tumour, malignant bone tumour, soft tissue sarcoma and germ cell tumour than in GHS controls. No difference was found for CCS with prior lymphoma. Previous radiotherapy of head or neck was associated with a smaller CRVE. Higher mean arterial blood pressure and intake of antihypertensive medication were associated with smaller CRAE.

**Conclusions:**

Retinal vasculature is altered in CCS leading to smaller retinal arteries and veins. Our finding indicates that childhood cancer and its treatment leads to systemic alterations of the microcirculation on both branches of the vasculature system. While the retinal venous vasculature is altered by radiotherapy, the lower vessel width of the arterial branch is associated with arterial hypertension.

## 1 INTRODUCTION

In the last decades, survival of childhood cancer has increased significantly due to new and optimized therapies leading to average overall survival of above 80% (Kaatsch et al., 2016). Adverse late effects in long‐term childhood cancer survivors (CCS) such as secondary cancer, endocrine disorders, impaired fertility and mental health and higher risk for cardiovascular diseases (CVD), therefore, gain in importance (Barton et al., 2013; Faber et al., 2018; Marchak et al., 2022; Mostoufi‐Moab et al., 2016). In CCS, CVD is the most frequent non‐neoplastic cause of premature mortality. In CCS over 60 years of age CVD is more frequent than subsequent primary neoplasms (Fidler et al., 2016). While several studies investigated cardiac and vascular toxicity with questionnaires and (hospital) administrative data (Gudmundsdottir et al., 2015; Mulrooney et al., 2009; Oeffinger et al., 2006; Olsen et al., 2014; van der Pal et al., 2012; van Waas et al., 2010), few studies have conducted a comprehensive investigation of the cardiovascular status (Faber et al., 2018; Hudson et al., 2013; Lipshultz et al., 2012; Mulrooney et al., 2016) with a particular lack of the microcirculation.

The retinal vasculature gives the unique opportunity to non‐invasively examine parts of the human microcirculation. Imaging modalities such as fundus photography allow to quantify the retinal vasculature. Retinal vessel parameters show associations to cardiovascular risk factors (Schuster et al., 2015; Wong et al., 2003). In addition, retinal arteriolar narrowing is reported to be a risk factor for future cardiovascular diseases (Wong, Klein, et al., 2004; Wong et al., 2005; Wong, Shankar, et al., 2004) and mortality (Wong, Knudtson et al., 2004).

The cardiac and vascular late sequelae in long‐term survivors of childhood cancer (CVSS)‐study was designed to incorporate both a detailed phenotyping of the cardiovascular status and an ophthalmological examination including fundus photography. To quantify alterations in the microcirculation in childhood cancer survivors (CCS), retinal vasculature measures of the arterial and venous branch were analysed and compared to data from a population‐based sample.

## 2 MATERIALS AND METHODS

### 2.1 Participants

The CVSS‐study is a retrospective registry‐based cohort study with a consecutive comprehensive examination at the study centre. German CCS were eligible for participation when diagnosed with a neoplasia prior to the age of 15 years according to the International Classification of Childhood Cancer (ICCC3). In addition, eligible criteria were suffering from childhood cancer between 1980 and 1990 and been registered at the German Childhood Cancer Registry, having survived more than 5 years after initial cancer diagnosis and been treated at one of 34 paediatric cancer centre taking part in the CVSS‐study. Further details of the CVSS‐study are described by Faber et al. (2018).

Among 2894 invited CCS, 1002 subjects took part in the examination between October 2013 and February 2016 at the study centre at the University Medical Center Mainz. The CVSS‐study participants underwent a standardized clinical examination at the Gutenberg Health Study (GHS) examination platform (Wild et al., 2012). All procedures were carried out in accordance with the Declaration of Helsinki and been approved by the ethics review committee of Rhineland‐Palatinate Chamber of Physicians. All participants gave written informed consent for study participation.

### 2.2 Medical data

A computer‐assisted personal interview (CAPI) was conducted to collect medical history data and, if available, medical records were retrieved. Cancer‐ and treatment‐related data were obtained from primary health records of former treating medical centres and/or individual therapy data at the respective study centres.

### 2.3 Assessment of cardiovascular risk factors

Blood pressure was measured with the OMRON HEM‐705CP II on the right arm in upright sitting position on a height‐adjustable chair with the back supported, legs uncrossed and feet on the ground, and with the lower arm resting on the table at heart level. The size of the cuff was chosen in relation to the width of the arm in every participant. The resting time before the first measurement was 3 min. Arterial hypertension was diagnosed if antihypertensive drugs were taken, or if the mean systolic blood pressure was at least 140 mmHg, or if the mean diastolic blood pressure was at least 90 mmHg, based on the mean of the second and third measurements after 8 and 11 min of rest. The definitions of other cardiovascular risk factors were previously described (Faber et al., 2018).

### 2.4 Assessment of ophthalmological parameters

For each study participant a comprehensive ophthalmological investigation was performed including fundus photography. In addition, objective refraction (Humphrey Automated Refractor/Keratometer (HARK) 599, Carl Zeiss Meditec AG, Jena, Germany) was performed and visual acuity was investigated. Non‐contact tonometry (Nidek NT‐2000, Nidek Co, Japan) was repeated three times to measure intraocular pressure.

### 2.5 Evaluation of retinal vasculature

Static retinal vessel analysis was performed by a trained grader (T.J.) using VesselMap II Software (Imedos Systems, Jena, Germany, Version 3.02. 2006) on 45° fundus photographs centred on the optic disc. The principles of static retinal vessel analysis have been previously described (Ponto et al., 2017). In brief, the optic nerve head is marked and the software creates an area of one half to one disk diameter distance around the optic disk. In this area, all retinal vessels are measured. Arterioles and venules are differentiated and selected manually (Figure S1). The retinal vessel equivalents, namely the central retinal arteriolar equivalent (CRAE), the central retinal venular equivalent (CRVE) and the arteriovenous ratio (AVR) were computed using the formula by Parr and Spears (1974) and Hubbard et al. (1999).

To assess intra‐ and inter‐observer reliability, all quantitative parameters (CRAE, CRVE and AVR) were assessed by the two graders in a subset of images and interclass correlation coefficients were computed. A random number generator was applied to randomly choose the photograph of the right or left eye for analysis. In case of missing photographs or insufficient image quality, the contralateral eye was chosen.

### 2.6 Comparative data from the Gutenberg health study

Retinal vessel characteristics of the CVSS‐study were compared to a sample of the GHS population as cardiovascular data and ophthalmological examinations were obtained in identical examinations. The GHS is a population‐based, prospective, observational, single‐centred cohort study including 15 010 study participants from the general population of Mainz and Mainz‐Bingen (Wild et al., 2012). Previously, normative data on retinal vessel characteristics based on this cohort were published (Ponto et al., 2017).

### 2.7 Statistical analysis

Descriptive statistics were computed for demographic, clinical and treatment parameters. Measures of retinal vessel characteristics (CRAE, CRVE and AVR) were computed according to ICCC3 diagnoses. For comparison with the GHS cohort, only the subset with an overlapping age range (35–48 years) was analysed.

Linear regression analysis was conducted to analyse the impact of childhood cancer on retinal vessel characteristics with adjustment on age, sex and spherical equivalent in model #1 and additional adjustment for cardiovascular risk factors (mean arterial blood pressure, intake of antihypertensive medication) in model #2. The impact of different ICCC3 diagnosis compared to control subjects were analysed. As sensitivity analysis, a 1:1 matching on age and sex between CVSS‐study and GHS was carried out. Within the CVSS‐study, the impact of chemotherapy and radiotherapy was further examined.

Statistical analyses were performed using Software R (version 3.4.0, The R Foundation for Statistical Computing, Vienna, Austria).

## 3 RESULTS

Among 1.002 examined participants in the CVSS‐study, the retinal vasculature of 837 individuals were analysed (Figure S2). This subset of the CVSS‐study comprised 379 (45.3%) female and 458 (54.7%) male participants with a mean age of 34.1 ± 5.6 years at examination and a mean age of 5.7 ± 4.3 years at initial childhood cancer diagnosis. The leading tumour entities were leukaemia (*n* = 364; 43.4%) and tumours of the central nervous system (*n* = 104; 12.4%). Other tumour entities were neuroblastoma (*n* = 88), renal tumour (*n* = 67), bone tumour (*n* = 66), soft tissue sarcoma (*n* = 60), germ cell tumour (*n* = 45) and others (*n* = 20; carcinoma, hepatic tumour and retinoblastoma). Antineoplastic treatment data was obtained for 91.7% (*n* = 768) of the CVSS‐study participants: the majority either received chemo‐ (*n* = 715) or radiotherapy (*n* = 446). Further demographic, diagnostic and treatment characteristics are presented in Table 1. The analysed study participants with retinal vasculature measurement did not differ from the rest of the CVSS‐study in a non‐responder analysis (Table S1). The characteristics of the GHS sample as control group (*n* = 1.667) is shown in Table S2.

**TABLE 1:** Demographic, diagnostic and treatment characteristics of CVSS‐study participants with available retinal measurements (*n* = 837).

CVSS	% (*n*)
Sex (female)	45.3 (379)
ICCC3 diagnosis	
Leukaemia	43.5 (364)
Central nervous system tumour	12.4 (104)
Neuroblastoma	10.5 (88)
Renal tumours	8.0 (67)
Soft tissue sarcomas	7.2 (60)
Malignant bone tumours	7.9 (66)
Germ cell tumours	5.4 (45)
Lymphoma	2.7 (23)
Others	2.4 (20)
Age at diagnosis (years)	
≤1	17.0 (142)
1 to ≤5	39.7 (332)
>5 to ≤8	13.9 (116)
>8 to ≤11	14.9 (125)
>11	14.6 (122)
Follow‐up time (years)	
<25	9.9 (83)
25 to <30	52.6 (440)
≥30	37.5 (314)
Treatment	
Chemotherapy	89.0 (715)
Radiation head/neck	84.5 (371)
Radiation other body organs	33.9 (148)
No chemotherapy, no radiation	7.9 (64)
Cardiovascular parameters	
Arterial hypertension	23.7 (198)
Intake of antihypertensive medication	8.4 (70)
Mean arterial blood pressure [mmHg]	94.4 ± 9.7

*Note*: Treatment data are available in 91.7% (*n* = 768). In the subcategory “others” are retinoblastoma, carcinoma and others summarized.

Abbreviations: CVSS, cardiac and vascular late sequelae in long‐term survivor of childhood cancer; ICCC3, International Classification of Childhood Cancer.

**FIGURE 1:**
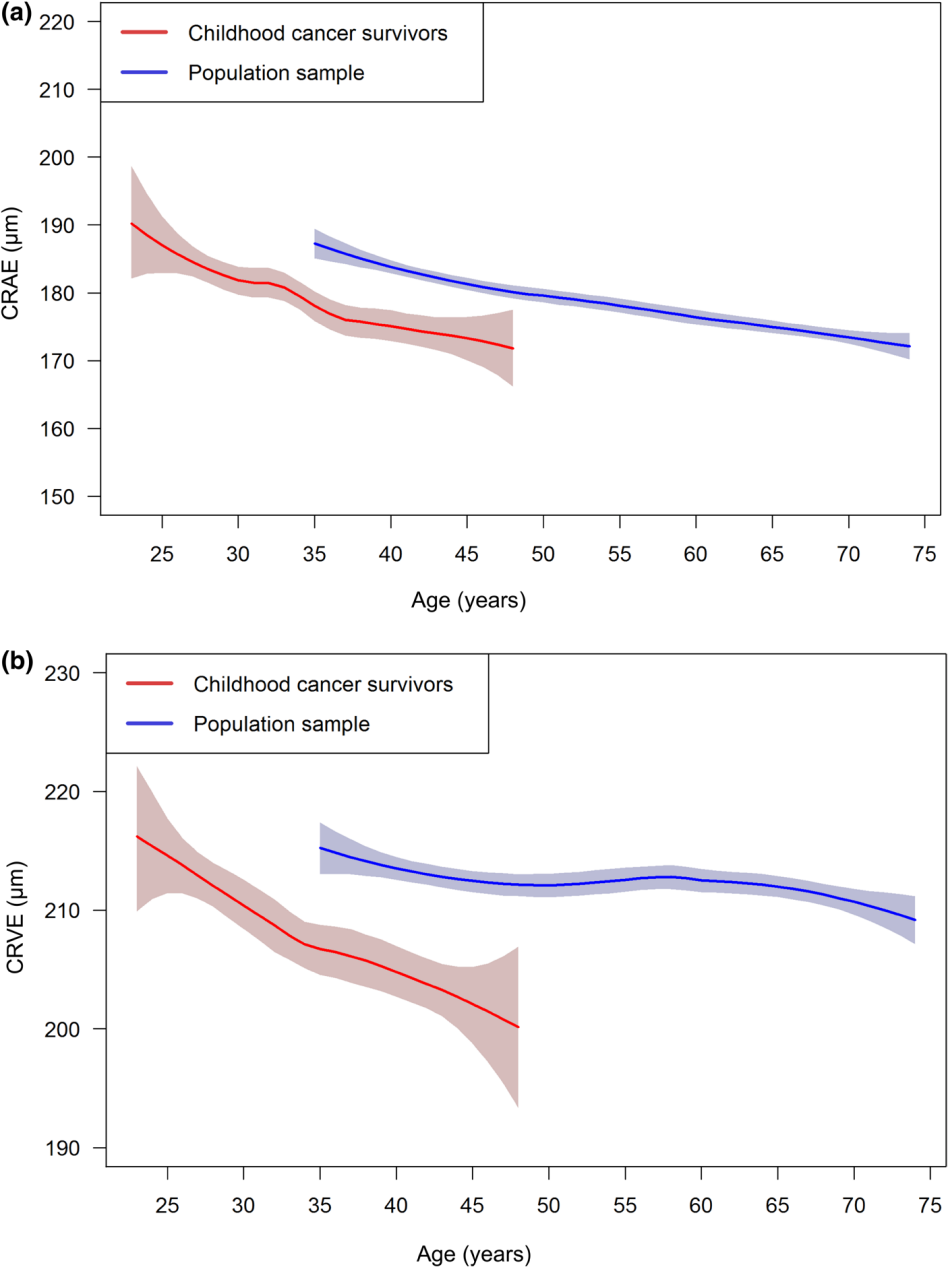
Comparison of characteristics of the retinal vasculature in (a) the arterial and (b) the venous branch stratified on childhood cancer diagnosis. The central retinal arteriolar equivalent (CRAE in μm) and the central retinal venular equivalent (CRVE in μm) was compared between study participants in the CVSS‐Study (*n* = 837) and the GHS (*n* = 1667). CVSS, cardiac and vascular late sequelae in long‐term survivor of childhood cancer; GHS, Gutenberg Health Study.

With respect to retinal vasculature characteristics, both central retinal arteriolar equivalent (CRAE) and central retinal venular equivalent (CRVE) was smaller in CCS than in GHS subjects (Figure 1). Linear regression analysis revealed that CRAE was 7.1 μm smaller in CCS than GHS subjects after adjustment for age, sex and spherical equivalent, and CRVE 7.7 μm, respectively (Table 2). Arteriovenous ratio (AVR) did not differ significantly between CCS and GHS subjects (*B* = 0; [−0.01; 0.01], *p* = 0.57).

**TABLE 2:** Associations of prior childhood cancer disease and retinal vasculature measures.

Prior childhood cancer	Model #1			Model #2		
	*B* estimate	95%‐CI	*p*‐value	*B* estimate	95%‐CI	*p*‐value
CRAE	−7.1	[−8.9; −5.3]	<0.0001	−6.5	[−8.2; −4.9]	<0.0001
CRVE	−7.7	[−9.4; −6.0]	<0.0001	−7.7	[−9.4; −6.0]	<0.0001
AVR	0.00	[−0.01; 0.01]	0.57	0.00	[−0.01; 0.01]	0.85

*Note*: Data from the CVSS‐study (*n* = 837) and GHS (*n* = 1.667). Linear regression analysis was computed with adjustment for age, sex and spherical equivalent in Model #1 and additional adjustment for mean arterial blood pressure and antihypertensive medication in Model #2. For CRAE, CRVE and AVR, a separate linear regression analysis was computed.

Abbreviations: AVR, arteriovenous ratio; CRAE, central retinal arteriolar equivalent; CRVE, central retinal venular equivalent; CVSS, cardiac and vascular late sequelae in long‐term survivor of childhood cancer; GHS, Gutenberg Health Study.

The sensitivity analysis incorporating a 1:1 sex‐ and age‐matching (CVSS and GHS *n* = 337) confirmed smaller CRAE in CCS (175 ± 17 μm vs. 183 ± 17 μm; *p* < 0.0001) and smaller CRVE in CCS (205 ± 16 μm vs. 212 ± 16 μm; *p* < 0.0001), but similar AVR (0.86 ± 0.08 vs. 0.86 ± 0.07; *p* = 0.18; Table S2). Intra‐ and interrater reliability was high: Intraclass correlation coefficient for CRAE and CRVE: 0.87 and AVR: 0.62.

Mean arterial blood pressure (*B* = −5.9/10 mmHg, 95%‐CI: [−6.5; −5.3], *p* < 0.0001) and intake of antihypertensive medication (*B* = −2.2; [−4.3; −0.1], *p* = 0.04) was associated with CRAE in the multivariable model. CRVE was slightly associated with mean arterial blood pressure: (*B* = −0.6 [−1.3; −0.02], *p* = 0.04) but not with intake of antihypertensive medication (*p* = 0.91). After adjusting for mean arterial blood pressure and intake of antihypertensive medication CRAE and CRVE remained smaller in CVSS than in GHS participants (Table 2).

**FIGURE 2:**
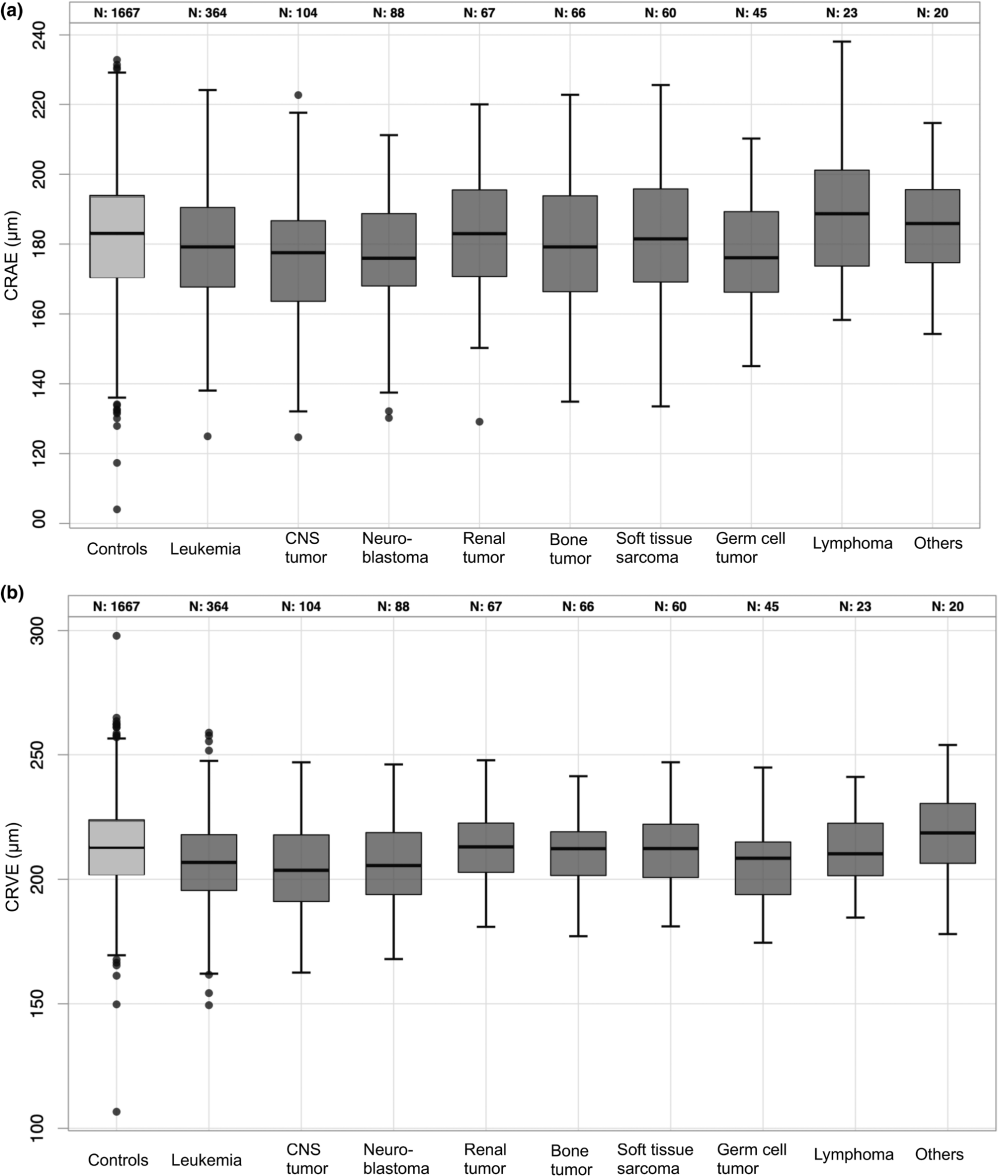
Comparison of characteristics of the retinal vasculature in (a) the arterial and (b) the venous branch stratified on childhood cancer diagnosis. The central retinal arteriolar equivalent (CRAE) and the central retinal venular equivalent (CRVE) was computed in the CVSS‐Study (*n* = 837) and stratified on diagnosis according to the International Classification of Childhood Cancer (ICCC3). CNS tumour, central nervous system tumour; CVSS, cardiac and vascular late sequelae in long‐term survivor of childhood cancer. Others: Including participants with prior carcinoma, hepatic tumour and carcinoma.

CCS previously having leukaemia, central nervous system tumour, neuroblastoma, renal tumour, malignant bone tumour, soft tissue sarcoma and germ cell tumour showed smaller CRAE and CRVE, while lymphoma and other tumour entities did not differ from the controls (Figure 2, Table 3). AVR did not differ significantly in any tumour group.

**TABLE 3:** Associations of entity of childhood cancer with retinal vasculature measures.

	CRAE			CRVE		
	*B* estimate	95%‐CI	*p*‐value	*B* estimate	95%‐CI	*p*‐value
Leukaemia	−6.5	[−8.6; −4.4]	<0.0001	−8.4	[−11; −6.3]	<0.0001
Central nervous system tumour	−8.5	[−12; −5.4]	<0.0001	−10	[−13; −7.1]	<0.0001
Neuroblastoma	−7.0	[−10; −3.7]	<0.0001	−7.3	[−11; −4.0]	<0.0001
Renal tumour	−5.6	[−9.5; −1.7]	0.005	−4.1	[−8.0; −0.2]	0.042
Malignant bone tumour	−8.6	[−13; −4.5]	<0.0001	−6.9	[−11; −2.8]	0.001
Soft tissue sarcoma	−6.0	[−10; −2.0]	0.004	−6.8	[−11; −2.8]	0.001
Germ cell tumour	−5.8	[−10; −1.4]	0.010	−6.7	[−11; −2.3]	0.003
Lymphoma	2.0	[−4.2; 8.2]	0.53	−3.8	[−10; 2.5]	0.24
Others	−4.6	[−11; 2.1]	0.18	2.8	[−4.0; 9.6]	0.41

*Note*: Data from the CVSS‐study (*n* = 837) and GHS (*n* = 1.667), GHS subjects are the reference (controls). Linear regression analysis was computed with adjustment for age, sex and spherical equivalent, mean arterial blood pressure and antihypertensive medication. For CRAE and CRVE, a separate linear regression analysis was computed. Others: including participants with prior carcinoma, hepatic tumour and carcinoma.

Abbreviations: CRAE, central retinal arteriolar equivalent; CRVE, central retinal venular equivalent; CVSS, cardiac and vascular late sequelae in long‐term survivor of childhood cancer.

With respect to treatment modality in the CVSS‐study, radiotherapy of head or neck (*B* = −3.6 [−5.8; −1.4]. *p* = 0.001) was associated with a smaller CRVE, but not with CRAE. Additionally, CRVE was more pronounced associated to radiation of other body parts, i.e. percutan or cardiac directed radiotherapy (*B* = −3.6 [−6.3; −0.8] *p* = 0.012) than CRAE (*B* = ‐3.0 [−5.8; −0.09] *p* = 0.044; Table 4). Chemotherapy was not associated with both CRAE and CRVE. Only detailed analysis of chemotherapy modalities revealed a positive association of ifosfamide with CRAE (*B* = 4.5 [1.3; 7.6] *p* = 0.005; Table S3).

**TABLE 4:** Associations of treatment status of childhood cancer with retinal vasculature measures.

	CRAE			CRVE		
	*B* estimate	95%‐CI	*p*‐value	*B* estimate	95%‐CI	*p*‐value
Chemotherapy (yes)	−0.69	[−2.9; 4.3]	0.71	1.4	[2.1; 4.8]	0.44
Radiation: head/neck (yes)	−0.32	[−2.6; 2.0]	0.78	−3.6	[−5.8; −1.4]	0.001
Radiation: other body organs (yes)	−3.0	[−5.8; −0.09]	0.044	−3.6	[−6.3; −0.8]	0.012

*Note*: Data from the CVSS‐study (*n* = 837). Linear regression analysis was computed with adjustment for age, sex and spherical equivalent, mean arterial blood pressure and antihypertensive medication. For CRAE and CRVE, a separate linear regression analysis was computed. Radiation of other body organs: cardiac directed or percutan radiotherapy.

Abbreviations: CRAE, central retinal arteriolar equivalent; CRVE, central retinal venular equivalent; CVSS, cardiac and vascular late sequelae in long‐term survivor of childhood cancer.

## 4 DISCUSSION

Retinal vasculature is altered in childhood cancer survivors leading to both smaller measures of retinal arteries and of retinal veins, as quantified by central retinal vessel equivalents.

Cardiovascular diseases are the most common cause of non‐cancer‐related death in childhood cancer survivors (Fidler et al., 2016). Faber et al. (2018) specifically demonstrated the increased prevalence of arterial hypertension and lipid metabolism disorders 15 years after childhood cancer therapy. High blood pressure in particular is one of the risk factors for cardiovascular diseases such as stroke or myocardial infarction and the associated increased mortality. Arterial hypertension also elevates the risk of diseases not primarily classified as cardiovascular diseases, such as dementia and chronic kidney disease (Zhou et al., 2021).

Fundus photographs allow to non‐invasive assess vascular changes of the microcirculation. Since early signs are difficult to objectify despite established classification systems (i.e. Wong‐Mitchell classification system (Wong & Mitchell, 2004)) and late retinopathy signs are rare nowadays (Tsukikawa & Stacey, 2020), more quantitative methods such as computer‐assisted static evaluation using fundus photographs have become established (Dong et al., 2022). In several population‐based studies, such as the North American “Multi‐Ethnic‐Study of Atherosclerosis” (MESA) examining 6237 participants aged 45–84 years of different ethnicity over 3.2 years, it was shown that a change in the retinal vessel width (decrease in the central artery equivalent) can even precede arterial hypertension, which is why static vessel analysis appears to be useful as a pre‐clinical surrogate parameter (Kawasaki et al., 2009). Numerous studies have demonstrated the relationship between reduced central artery equivalent and arterial hypertension (Ikram et al., 2006; Lona et al., 2020; Xue et al., 2023). The Gutenberg Health Study also previously reported a reduced central artery equivalent in persons with untreated or insufficiently treated hypertension (Ponto et al., 2017). Even in children and adolescents, an increase in blood pressure is related to a significant decrease in the central artery equivalent, but not to the central vein equivalent, as previously reported in a meta‐analysis (Köchli et al., 2018).

But not only blood pressure levels can significantly impact the retinal vessel calibre. Reportedly, age, alcohol consumption and obesity promote narrowing of retinal arterial vessels, whereas diabetes and smoking are associated with wider arterial vessels. In fact, the central venous equivalent appears not to be influenced by blood pressure but by a variety of other factors. Studies report wider retinal venous vessels with younger age, endothelial dysfunction, diabetes, systemic inflammation, smoking, dyslipidaemia and obesity (Sun et al., 2009; Wong et al., 2006).

We found an alteration of the retinal vasculature in CCS compared to an age‐ and sex‐matched cohort from the population‐based GHS. Both CRAE and CRVE were significantly smaller in CCS. AVR, as a quotient of CRAE and CRVE, did not change significantly. These findings persisted after adjusting for antihypertensive treatment and mean arterial blood pressure, which are known to influence CRAE. However, not only arterial hypertension but other metabolic factors like dyslipidaemia, obesity or endothelial dysfunction are known to be more prevalent in CCS which were shown to influence the retinal vasculature (Faber et al., 2018; Siviero‐Miachon et al., 2008; Sun et al., 2009; Wong et al., 2006).

Furthermore, Arnold et al. (2021) reported an increased arterial stiffness in CCS compared to the general population after adjusting for cardiovascular risk factors. The increased arterial stiffness might be a result of endothelial injury and induction of chronic oxidative stress by prior treatment with chemotherapy and radiation (Svilaas et al., 2016; Zhao et al., 2007). We did not find an association of prior chemotherapy and retinal vasculature measurements, but prior radiation of head and neck had a significant influence on CRVE and radiation of other body parts (i.e. cardiac and percutan) both on CRAE and CRVE. Radiation is known to trigger fibrogenesis in a dose dependent manner resulting in radiation fibrosis leading to a loss of tissue and vascular compliance (Yarnold & Vozenin Brotons, 2010). This could explain smaller CRAE und CRVE in treated patients.

Interestingly, Arnold et al. (2021) reported that CCS without chemotherapy or radiation showed increased arterial stiffness as well and Lipshultz et al. (2012) reported cardiovascular abnormalities, systemic inflammation and increased risk of atherosclerotic disease in CCS without cardiotoxic treatment (anthracyclines and cardiac radiation).

Additionally, we detected differences between the tumour entities. All tumour entities, except lymphoma and others (carcinoma, hepatic tumours and retinoblastoma; *n* = 20, probably the group was too small and heterogenic for statistical analysis), were associated with smaller CRAE and CRVE. Arnold et al. (2021) found and increased arterial stiffness in all groups; however, the least pronounced in the lymphoma subgroup, suggesting a possible link. Therefore, it is likely that the detected alteration of the retinal vasculature is a result of the complex interplay of the malignant disease itself and neuro‐ and vasculotoxic effects of the treatment.

There are some limitations of our study. First, recruitment of the GHS is based on the region of Mainz and Mainz‐Bingen, while the CVSS‐study recruited from Germany. Since both samples are population‐based, and as treatment patterns for childhood cancer are consistent for Germany, the regional restraints are less prominent and the results can be transferred to the German population. Childhood Cancer Survivors have a selection bias by definition: early mortality will lead to a larger proportion of better treatable cancer types in such studies. Furthermore, subjects with severe physical or mental impairment are less likely to participate. Imaging of the retinal vasculatures and quantification of retinal vessel parameters was not sufficiently possible in each study participant. Nevertheless, item‐non‐responder analysis revealed that subjects without retinal vessel measurement were not different to those being included in the analysis. Previous reports state high reliability measures (Ponto et al., 2017), similar to ours.

In summary, our findings indicate that childhood cancer and its treatment lead to systemic alterations of the microcirculation on both branches of the vasculature system. While the retinal arterial vasculature is associated with cardiovascular diseases such as arterial hypertension that show a higher prevalence in CCS, the venous branch shows alterations due to radiotherapy. Examination of the retina is cost effective and non‐invasive and might help to monitor this vulnerable patient group in the future since a link between systemic cardiovascular risk factors and increased mortality in patients with altered retinal vasculature is known.

## FUNDING INFORMATION

Schuster AK received technical support by Heidelberg Engineering (Heidelberg, Germany) and financial support by Novartis and Bayer Vital; Pfeiffer N: receives financial support and grants by Novartis, Ivantis, Santen, Thea, Boehringer Ingelheim Deutschland GmbH & Co. KG, Alcon, Sanoculis; Wild PS: received outside the submitted work, consulting fees from Astra Zeneca, research funding from Bayer AG, research funding, consulting and lecturing fees from Bayer Health Care, lecturing fees from Bristol Myers Squibb, research funding and consulting fees from Boehringer Ingelheim, research funding and consulting fees from Daiichi Sankyo Europe, consulting fees and non‐financial support from Diasorin, non‐financial research support from I.E.M., research funding and consulting fees from Novartis Pharma, lecturing fees from Pfizer Pharma, non‐financial grants from Philips Medical Systems, research funding and consulting fees from Sanofi‐Aventis; all other authors: none.

## CONFLICT OF INTEREST STATEMENT

None for all authors.

## Supporting information


Figure S1.

Figure S2.

Table S1.
Table S2.
Table S3.
