# Supplementary figures and images for: Altered retinal vasculature in childhood cancer survivors: Data from the German CVSS‐study
Source: Acta Ophthalmol. 2025 Jan 23;103(4):e231–9. doi: 10.1111/aos.17438 (PMC12069964; doi:10.1111/aos.17438)

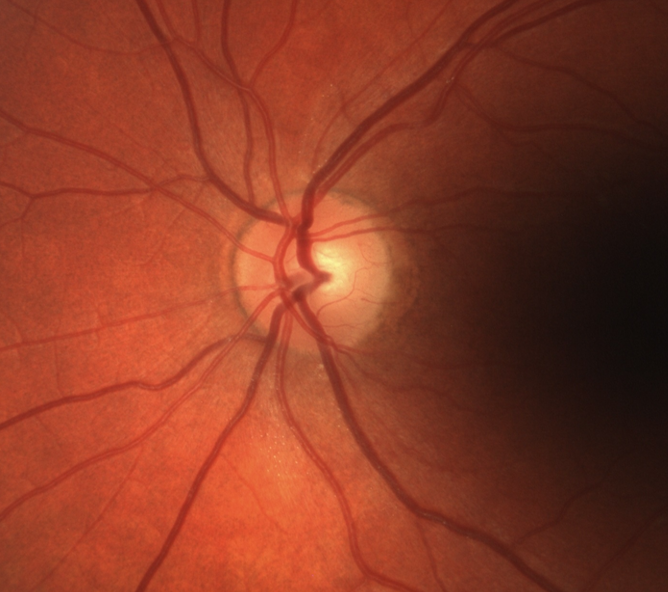

Supplement: Supplementary file 1 — Figure S1. [file AOS-103-e231-s001.zip › Figure S1a.png]

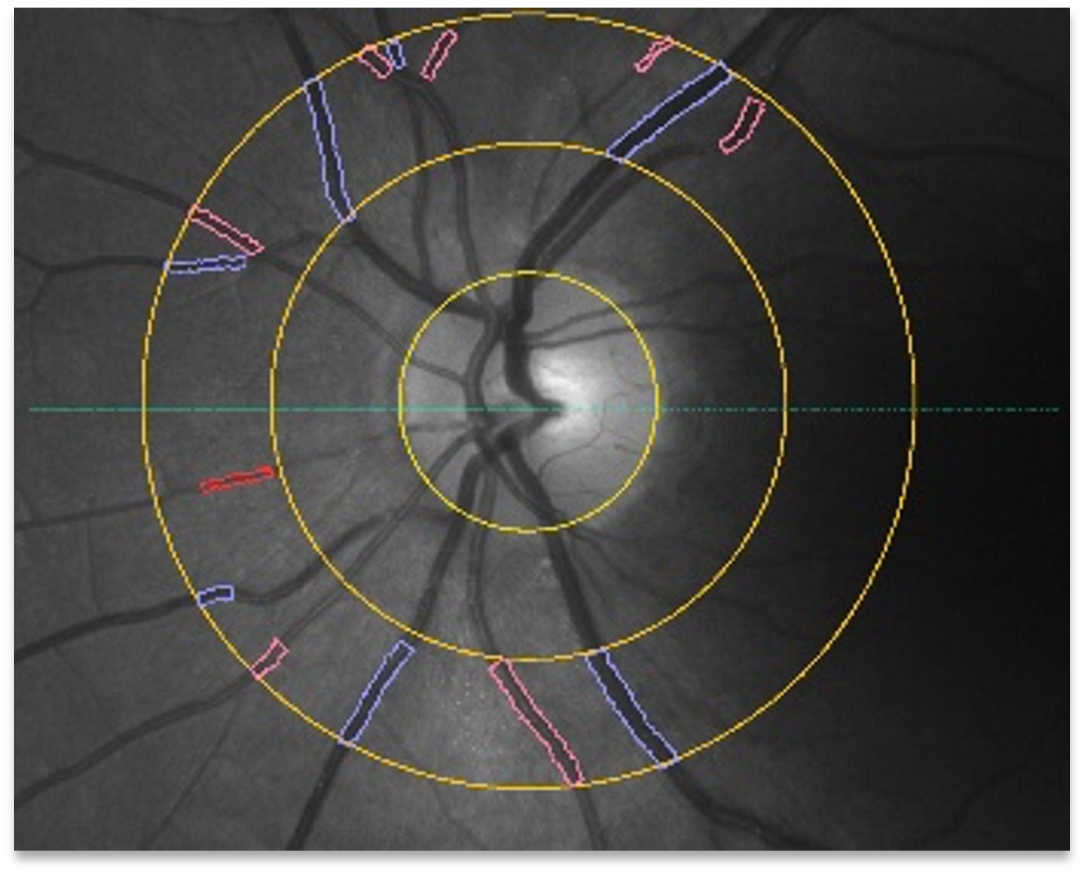

Supplement: Supplementary file 1 — Figure S1. [file AOS-103-e231-s001.zip › Figure S1b.png]

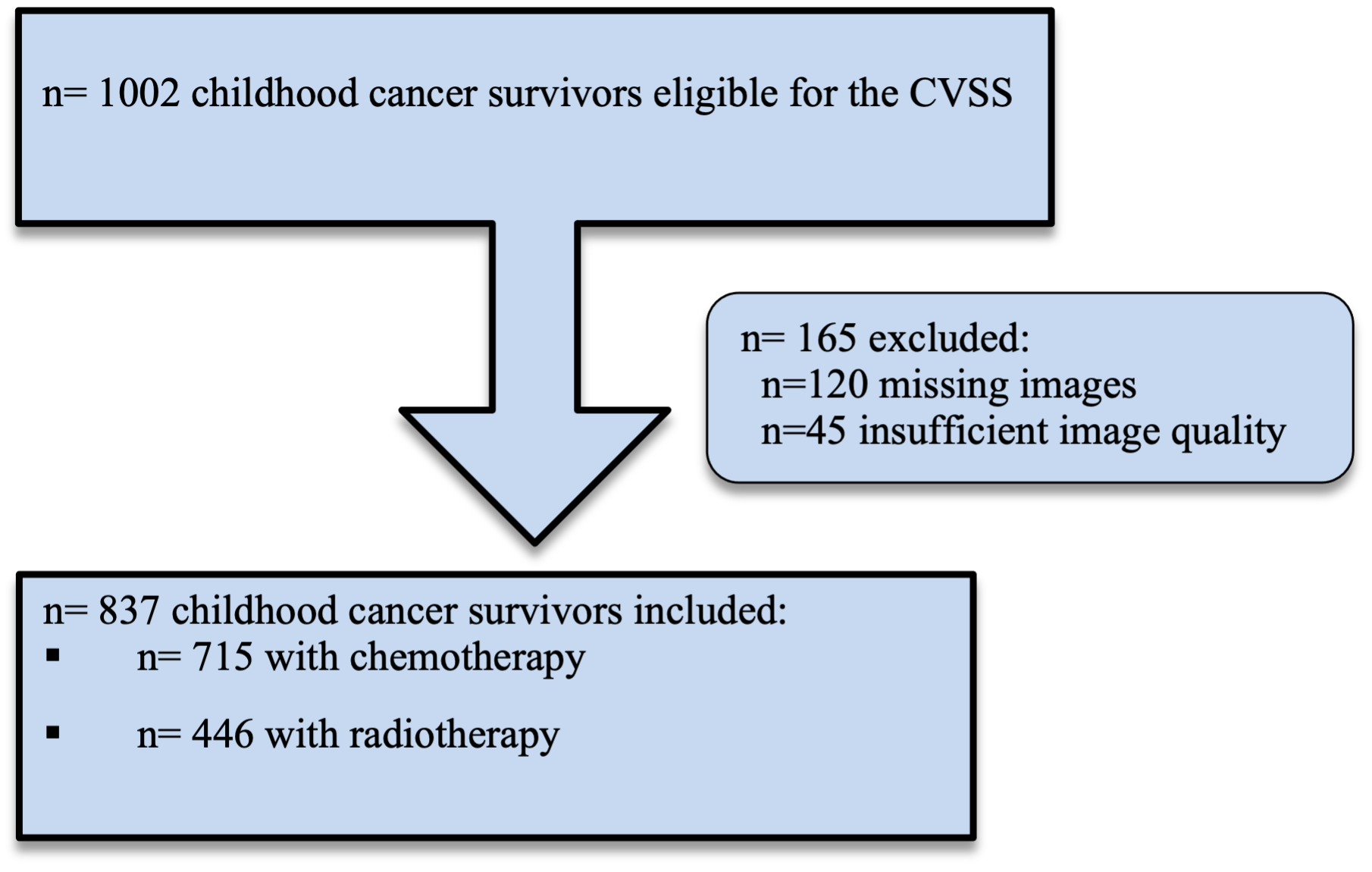

Supplement: Supplementary file 2 — Figure S2. [file AOS-103-e231-s002.png]
