# Supplementary material for: Altered retinal vasculature in childhood cancer survivors: Data from the German CVSS‐study
Source: Acta Ophthalmol. 2025 Jan 23;103(4):e231–9. doi: 10.1111/aos.17438 (PMC12069964; doi:10.1111/aos.17438)
Supplement: Supplementary file 3 — Table S1. Table S2. Table S3. [file AOS-103-e231-s003.docx]

**Table S1: Item-Non-Responder analysis in the CVSS-study (n=1,002) with respect to retinal vasculature measurement.**

| CVSS-study | | |
| --- | --- | --- |
|  | Responder  83.5% (837) | Non-Responder  16.5% (165) |
| Sex (female (%)) | 45.3% (379) | 46.7% (77) |
| Age (years)  Age of disease onset (years) | 34.1 ± 5.6  5.7 ± 4.3 | 34.6 ± 5.5  5.9 ± 4.2 |
| Follow-up time (years) |  |  |
| - <25 | 9.9% (83) | 10.3% (17) |
| - 25 - <30 | 52.6% (440) | 52.1% (86) |
| - ≥30 | 37.5% (314) | 37.6% (62) |
| Treatment (yes in %) | | |
| - Chemotherapy | 89.0% (715) | 87.8% (137) |
| - Radiotherapy | 56.9% (446) | 61.8% (94) |
| - Percutaneous radiotherapy | 56.4% (442) | 60.5% (92) |
| - Irradiation of head and/or   neck   - Irradiation of other body   regions   - No therapy | 84.5% (371)  33.9% (148)  7.9% (64) | 87.9% (80)  38.0% (35)  6.3% (10) |
| Cardiovascular parameters | | |
| - Arterial hypertension | 23.7% (198) | 26.1% (43) |
| - Intake of antihypertensive medication | 8.4% (70) | 7.9% (13) |
| - Mean arterial blood pressure (mmHg) | 94.4 ± 9.7 | 94.3 ± 10.7 |

CVSS: cardiac and vascular late sequelae in long-term survivor of childhood cancer.

**Table S2: Demographic characteristics of GHS sample included as control group to the CVSS-study (n=1667) and GHS sample included in 1:1 matched control group for the sensitivity analysis (n=337)**

| Variable | **GHS (n=1667)** | **CVSS**  **(n=1002)** | **p-value** | **GHS**  **(n=377)** | **CVSS**  **(n=377)** | **p-value** |
| --- | --- | --- | --- | --- | --- | --- |
| **sex (female %)** | 50.0% (833) | 45.3% (379) | **0.028** | 43.3% (165) | 43.3% (165) | 1.00 |
| **Age (years)** | 43.4±4.3 | 34.1±5.6 | **<0.0001** | 39.4±3.4 | 39.2±3.6 | 0.51 |
| Ophthalmologic parameters | | | | | | |
| **CRAE [µm]*^3^** | 182±17 | 179±18 | **0.00014** | 183±17 | 175±17 | **< 0.0001** |
| **CRVE [µm]*^4^** | 213±17 | 208±17 | **<0.0001** | 212±16 | 205±16 | **< 0.0001** |
| **AVR*^5^** | 0.86±0.07 | 0.86±0.07 | **0.037** | 0.86± 0.07 | 0.86± 0.08 | 0.18 |
| **Intraocular pressure [mmHg]** | 13.86±2.59 | 14.40±2.77 | **<0.0001** | 13.87± 2.62 | 14.22±2.71 | 0.069 |
| **Spherical equivalent [dpt]** | -0.56 (-1.81/0.06) | -0.81 (-1.94/-0.25) | **<0.0001** | -0.62 (-1.81/-0.12) | -0.87 (-2.50/-0.25) | **0.027** |
| **Self-reported cataract** | 0.3% (5) | 1.8% (15) | **0.00016** | 0.3% (1) | 1.9% (7) | 0.069 |
| **Self-reported glaucoma** | 0.4% (7) | 0.7% (6) | 0.38 | 0.5% (2) | 1.3% (5) | 0.45 |
| **Self-reported dry eye** | 0% (0) | 0.1% (1) | 0.33 | 0% (0) | 0% (0) | 1.00 |
| Systemic parameters | | | | | | |
| **Hypertension** | 28.1% (469) | 23.7% (198) | 0.019 | 21.5% (81) | 32.2% (121) | **0.0010** |
| **SBP [mmHg] ^*6^** | 125.1±14.2 | 123.0±12.9 | 0.00037 | 123.8±13.4 | 124.1±13.4 | 0.77 |
| **DBP [mmHg]*^7^** | 82.3±9.1 | 80.1±9.1 | **<0.0001** | 81.3±8.8 | 81.7±9.4 | 0.54 |
| **MAP [mmHg]*^8^** | 96.5±10.3 | 94.4±9.7 | **<0.0001** | 95.5±9.8 | 95.9±10.2 | 0.61 |
| **Antihypertensive drugs** | 8.6% (143) | 8.4% (70) | 0.94 | 5.3% (20) | 11.9% (45) | **0.0017** |
| Abbreviation: CVSS*^1^**^=^ Cardiac and vascular late sequelae in long-term survivors of childhood cancer-Study;** GHS*^2^ **= Gutenberg Health Study, Baseline Examination** CRAE*^3^ **=** central retinal arteriolar equivalent**;** CRVE*^4^ **=** central retinal venular equivalent**;** AVR*^5^**=** arteriovenous ratio**;** SBD**^*6^= systolic blood pressure;** DBP***^7^= diastolic blood pressure;** MAP***^8^= mean arterial blood pressure;** | | | | | | |

**Table S3: Detailed analysis of associations of treatment status of childhood cancer with retinal vasculature measures**

|  | **CRAE** | | | **CRVE** | | |
| --- | --- | --- | --- | --- | --- | --- |
|  | **B-estimate** | **95% CI** | **p-value** | **B-estimate** | **95% CI** | **p-value** |
| Anthracyclines (yes) | 1.1 | [-3.2; 5.3] | 0.63 | 2.1 | [-2.1; 6.2] | 0.33 |
| Cyclophosphamide (yes) | -3.1 | [-6.8; 0.57] | 0.098 | -2.7 | [-6.3; 0.82] | 0.13 |
| Ifosfamide (yes) | 4.5 | [1.3; 7.6] | **0.0054** | 0.84 | [-2.2; 3.9] | 0.59 |
| Vincaalcaloids (yes) | -0.24 | [-3.6; 3.1] | 0.89 | -1.5 | [-4.8; 1.7] | 0.36 |
| High dose steroids (yes) | 2.0 | [-2.6; 6.6] | 0.40 | -1.3 | [-5.8; 3.2] | 0.57 |
| Platine derivate (yes) | 1.2 | [-2.8; 5.2] | 0.56 | 2.2 | [-1.7; 6.1] | 0.28 |
| Methotrexate (yes) | -1.6 | [-5.8; 2.6] | 0.45 | 0.78 | [-3.3; 4.8] | 0.70 |
| Asparaginase (yes) | 1.2 | [-3.0; 5.4] | 0.58 | 2.8 | [-1.3; 6.9] | 0.18 |
| Sex (Women) | 0.86 | [-1.4; 3.1] | 0.45 | 0.70 | [-1.5; 2.9] | 0.53 |
| Age (years) | -0.23 | [-0.61; 0.14] | 0.22 | -0.22 | [-0.58; 0.15] | 0.24 |
| Spherical equivalent (D) | 2.5 | [2.0; 3.1] | **<0.0001** | 3.0 | [2.5; 3.5] | **<0.0001** |
| Age of disease onset (years) | -0.0085 | [-0.48; 0.46] | 0.97 | -0.25 | [-0.71; 0.21] | 0.28 |
| Mean arterial blood pressure (mmHg) | -0.62 | [-0.74; -0.51] | **<0.0001** | -0.089 | [-0.20; 0.024] | 0.12 |
| Antihypertensiva (yes) | -3.3 | [-7.3; 0.70] | 0.11 | -0.65 | [-4.5; 3.2] | 0.74 |

CRAE: central retinal arteriolar equivalent. CRVE: central retinal venular equivalent. CVSS: cardiac and vascular late sequelae in long-term survivor of childhood cancer.
